# The Story of the Insane from Year to Year

**Published:** 1903-09-19

**Authors:** 


### THE STORY OF THE INSANE FROM YEAR TO YEAR.

Sixteenth Annual Series.

### THE LEICESTER BOROUGH ASYLUM.

On January 1st this asylum contained 797 patients. During the year 164 patients were admitted for the first time, and 33 were readmitted. Of the total number under treatment 68 were discharged recovered; 4 were discharged relieved, 107 not improved, and 75 died. The total number under treatment was 985, and the average number resident was 791. Calculated on the admissions the recoveries stand at 46·2 per cent.; and calculated on the average number resident the deaths stand at 9·4. The recovery rate is rather above the average, and the death-rate is about the average of similar institutions. Of the year's deaths 30 were caused by cerebral and spinal diseases, 3 by pneumonia, and 8 by consumption. Of the admissions the insanity was put down to various moral causes in 18 instances, and of the physical causes, hereditary influence stands first with 34, previous attacks comes next with 20, and intemperance in drink was the cause in 17. About 370 of the patients are able to be employed at some useful occupation. No patient was subjected to mechanical restraint of any kind, and only a few were secluded for short periods. The head male attendant died of consumption, and a charge nurse and a laundry-maid obtained pensions during the year. Both were in ill-health, and one has since died. In connection with this subject it may be mentioned that a pensions scheme has been framed and awaits approval by the council. This asylum having more accommodation than is required for the borough, receives patients from other districts, and contracts are in force with Oxford, Cardiff, and Glamorgan. The committee say they "have pleasure in again recording their appreciation of the highly satisfactory manner in which Dr. Finch and the officers have discharged their respective duties." The weekly cost of maintenance is a little over 11s.

### JOINT COUNTIES' ASYLUM, CARMARTHEN.

At the beginning of the year there were 648 patients in residence. There were during the year 109 first admissions, 13 readmissions, 32 discharged recovered, 8 discharged relieved, 15 not improved, and 63 died. The average number resident was 646, and the total number under treatment was 770. The recoveries stand at 27·5 per cent. of the admissions, and the deaths at 8·18 per cent. of the average number resident. Of the deaths 18 were caused by cerebral and spinal diseases, 7 by pneumonia, and 16 by consumption. Turning to Table X. we find that in 27 cases the insanity was due to such moral causes as domestic trouble, shock, and adverse circumstances; and that among the physical causes hereditary influence produced 32 of the year's admissions and drink 22. The asylum is full, but if 33 out-county patients were to be discharged, there would be accommodation for the joint counties for between five and ten years. If the asylum visitors are wise they will employ this time in adding to their asylum. There is no greater extravagance than waiting until every bed is occupied before making additions. This is shown in their present report. The charge for out-county patients is 13s. a week, their own cost is 9s., so that for everyone received the joint counties ratepayer is gaining 4s. a week. Reverse this process and the ratepayer would lose 4s. a head per week. We have already stated that 16 of the deaths were caused by consumption, being 28·6 per cent. of the total number of deaths. A new infectious hospital is being built, and it has been suggested that the old one should be used as a sanatorium for consumption, and if reasonably suitable for this purpose, it is hard to see how Dr. Goodall could apply it to anything more useful. He states that "the segregation of cases of consumption is highly desirable, otherwise they act as foci of contagion." Dr. Goodall has a paragraph on the boarding-out system of treating the harmless insane, and he thinks they could be as economically maintained in private families as in asylums where the maintenance rate of such asylums is up to the average. This is no doubt true, even where the rate is a little under the average; but the system, although proved to be successful in Scotland has never taken root in England; and we doubt whether it will ever be carried out to an extent sufficient to sensibly reduce the numbers of our asylum population. The improvement of our workhouse wards for imbeciles, and a capitation grant to the guardians for all suitable cases kept therein would be more serviceable. When speaking of treatment, Dr. Goodall refers to electric baths, and says, also, that the wet-sheet pack is much extolled and is more used abroad than in England, where its use "is limited owing to regulations as to mechanical restraint." We regret to hear that such regulations should have interfered with so efficient a means of treatment; and we would ask whether the Commissioners have ever been approached with a view to their removing such restriction? We are glad to hear that the Visitors have at present under consideration the adoption of some scheme for pensioning the members of their staff, as we were under the impression that this was one of the asylums where pensions were discouraged. It is, however, hardly possible that even the most favourable pensions scheme can provide sufficient pension for an ordinary attendant or nurse to live on comfortably. To save money out of the wages given is always difficult; but a small fixed sum per quarter could be set aside, and we strongly advise every attendant and nurse to join the Royal National Pension Fund. To us it is a mystery how any attendant or nurse can be induced to enter an asylum where pensions are not granted, and we believe that the best members of the staff will always be more inclined to look upon the asylum as their home when these pensions will certainly come to them in old age. The weekly cost per head is 9s.; out-counties pay from 13s. to 14s.; and private patients from 10s. to 40s.

### WESTERN COUNTIES ASYLUM FOR IDIOTS, AT STARCROSS, DEVON.

On January 1 this institution contained 269 patients, and 46 were admitted during the year. The discharges were 44, classified as follows:—Much improved 16, moderately improved 22, not improved 6. There was only one death. The asylum is really a training school for feeble-minded children, and consequently none of the statistics can be brought into comparison with those of our county or borough asylums. We note that eight of those discharged had improved so much that their friends thought them capable of contributing to their maintenance. Others were discharged either because they were not susceptible of improvement or because they had derived all the benefit they were capable of. In the training of these children a large amount of patience is required on the part of the teacher, and it must be gratifying to them to see that satisfactory progress is made by some of the pupils. Mr. Locke, the superintendent, states that he gives great prominence to industrial training because better results can be obtained in this direction than in the more purely educational lessons, a statement which is quite easily understood. Many of his pupils competed at the Arts and Crafts Exhibition at Reigate, and were successful in obtaining 35 prizes. The income is derived from various sources, but chiefly from the payments made by private patients, and by cases sent in by the Boards of Guardians.

